# Successful management of malignant colovesical fistula using covered colonic self-expanding metallic stent: a case report

**DOI:** 10.1186/s40792-023-01784-8

**Published:** 2023-11-21

**Authors:** Goro Takahashi, Akihisa Matsuda, Takeshi Yamada, Kay Uehara, Seiichi Shinji, Yasuyuki Yokoyama, Takuma Iwai, Kohki Takeda, Sho Kuriyama, Toshimitsu Miyasaka, Shintaro Kanaka, Tai Terayachi, Tetsuya Okino, Hiroshi Yoshida

**Affiliations:** Department of Gastrointestinal and Hepato-Biliary-Pancreatic Surgery, Nippon Medical School Hospital, Tokyo, 113-8603 Japan

**Keywords:** Colovesical fistula, Covered stent, Self-expanding metallic stent

## Abstract

**Background:**

A colovesical fistula (CVF) is commonly treated by resection of the intestine containing the fistula or creation of a defunctioning stoma. We herein report a case of successful fistula closure and avoidance of colostomy after placement of a covered colonic self-expanding metallic stent (SEMS) as a palliative treatment for a malignant CVF.

**Case presentation:**

A 75-year-old man undergoing infusional 5-fluorouracil and irinotecan chemotherapy plus bevacizumab for recurrent peritoneal dissemination of rectal cancer was admitted to our hospital because of fecaluria with a high-grade fever. Blood tests showed a moderate inflammatory reaction (white blood cell count, 9200/mm^3^; C-reactive protein, 11.03 mg/dL; procalcitonin, 1.33 ng/mL). Urinary sediment examination showed severe bacteriuria. Abdominal contrast-enhanced computed tomography showed intravesical gas, thickening of the posterior wall of the bladder, and irregular thickening of the sigmoid colon wall contiguous with the posterior bladder wall. Magnetic resonance imaging (MRI) clearly showed a fistula between the bladder and sigmoid colon. Colonoscopy revealed a circumferential malignant stricture 15 cm from the anal verge, and a fistula to the bladder was identified by water-soluble contrast medium. We diagnosed a complicated urinary tract infection (UTI) associated with a CVF due to peritoneal dissemination and started empirical treatment with sulbactam/ampicillin. Given the absence of active inflammatory findings around the fistula on MRI and the patient’s physical frailty, we decided to place a covered SEMS to close the fistula. Under fluoroscopic and endoscopic guidance, a covered colonic SEMS of 80-mm length and 20-mm diameter was successfully deployed, and the fistula was sealed immediately after placement. Urine culture on day 3 after stenting was negative for bacteria, and a contrast study on day 5 showed no fistula. The patient was discharged home on day 6 with no complications. The UTI did not recur for 4 months after discharge.

**Conclusions:**

A covered colonic SEMS was useful for sealing a malignant CVF in a patient unfit for surgery, and MRI was valuable to determine the status of the fistula. A covered colonic SEMS could be an alternative to surgical treatment for CVFs in patients who require palliative care.

## Background

Colovesical fistulas (CVFs) usually cause recurrent urinary tract infections (UTIs) and significantly reduce patients’ quality of life [1]. Therefore, patients are commonly treated by resection of the intestine containing the fistula or creation of a defunctioning stoma [1, 2]. However, patients with a malignant CVF usually present in the advanced stages of their cancer, when radical surgery or even stoma creation may not be indicated [3, 4]. We herein report a case of a CVF caused by recurrent peritoneal dissemination after rectal cancer surgery. A covered colonic self-expanding metallic stent (SEMS) was placed to close the fistula, finally achieving control of the patient’s UTI and avoiding stoma creation.

## Case presentation

**Fig. 1:**
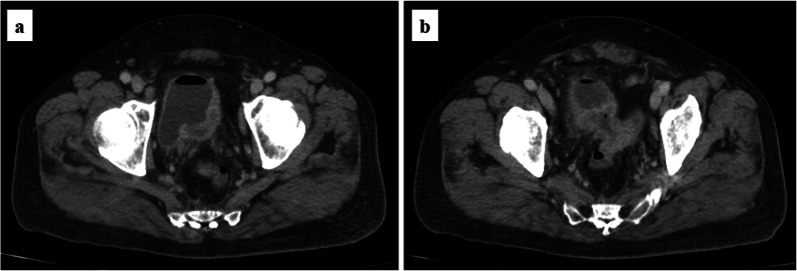
Computed tomography findings. **a** Postcontrast image showing intravesical gas and thickening of the posterior wall of the bladder. **b** Irregular thickening of the sigmoid colon wall contiguous with the posterior wall of the bladder

**Fig. 2:**
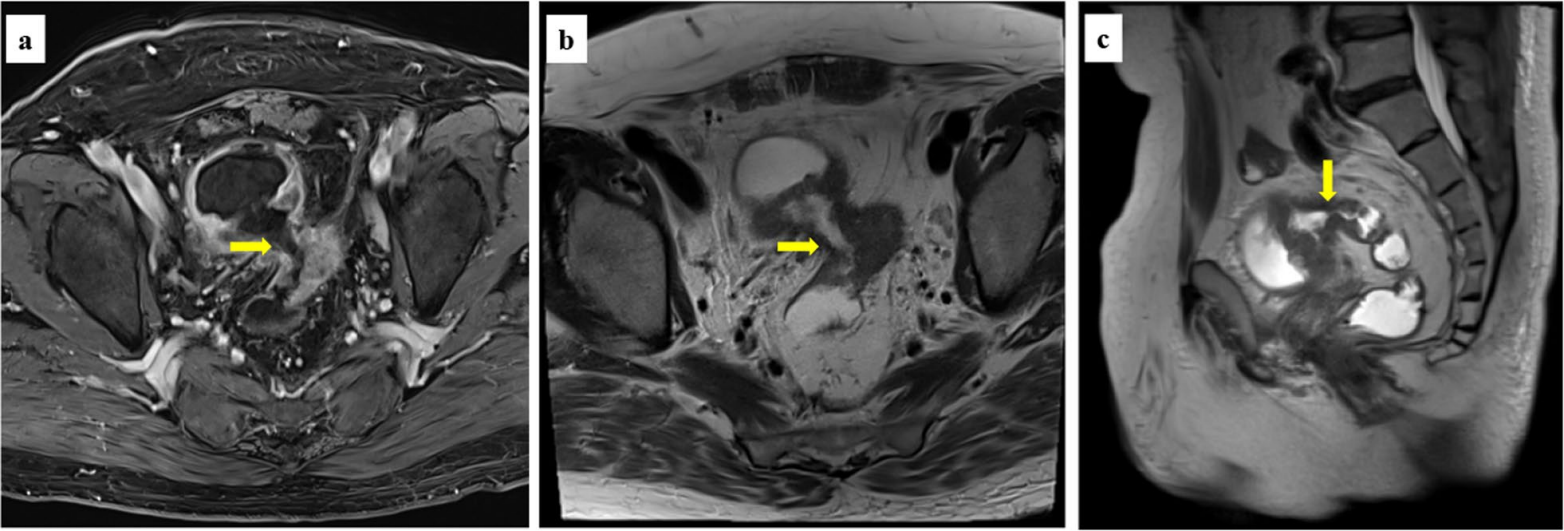
Magnetic resonance imaging clearly showed the fistula between the bladder and sigmoid colon. **a** T1-weighted image. **b**, **c** T2-weighted images

**Fig. 3:**
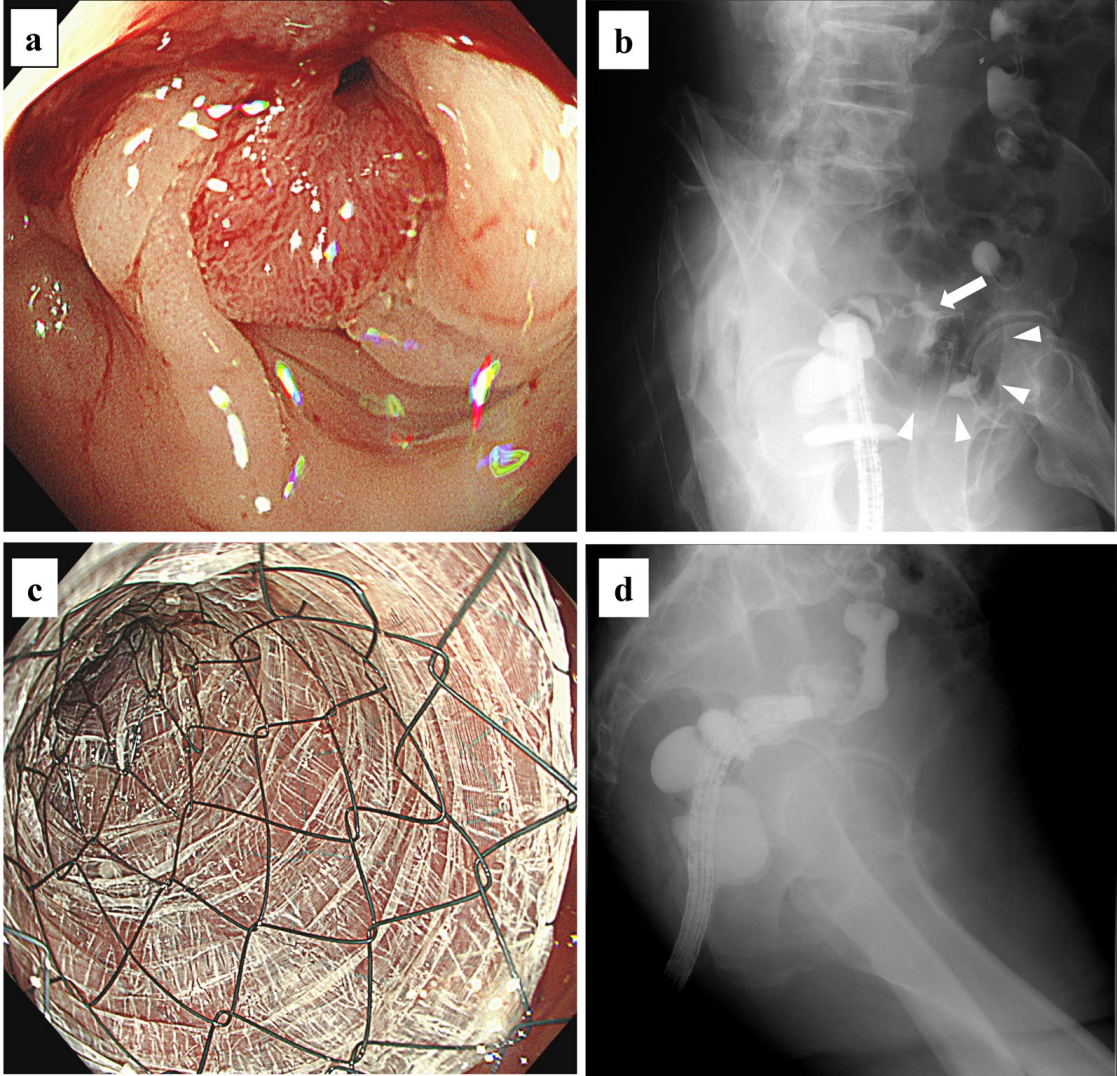
Fistula closure using a covered colonic self-expanding metallic stent. **a** Colonoscopy showed a circumferential malignant stricture 15 cm from the anal verge. **b** Colovesical fistula was delineated by water-soluble contrast medium (arrow). The arrowhead indicates the bladder. **c**, **d** Complete sealing of the colovesical fistula using a covered stent (80 mm long and 20 mm in diameter)

A 75-year-old man undergoing infusional 5-fluorouracil and irinotecan chemotherapy plus bevacizumab (BV) for recurrent peritoneal dissemination and a metastatic lung tumor from rectal cancer was admitted to our hospital because of pneumaturia and fecaluria with a high-grade fever. Blood tests showed a moderate inflammatory reaction (white blood cell count, 9200/mm^3^; C-reactive protein, 11.03 mg/dL; procalcitonin, 1.33 ng/mL). Urinary sediment examination showed severe bacteriuria (bacteria, 3 + ; white blood cells, > 100/high-power field). Abdominal contrast-enhanced computed tomography showed intravesical gas, thickening of the posterior wall of the bladder (Fig. 1a), and thickening of the sigmoid colon wall contiguous with the posterior bladder wall (Fig. 1b). Magnetic resonance imaging (MRI) clearly showed a fistula between the bladder and sigmoid colon (Fig. 2). Colonoscopy revealed a circumferential malignant stricture 15 cm from the anal verge (Fig. 3a), and a fistula to the bladder was identified by water-soluble contrast medium (Fig. 3b). We diagnosed a complicated UTI associated with a CVF due to peritoneal dissemination and started empirical treatment with sulbactam/ampicillin after drawing blood for culture.

Given the absence of active inflammatory findings around the fistula on MRI and the patient’s physical frailty, we decided to place a covered SEMS to the stricture to seal the fistula and relieve the stenosis after obtaining informed consent. Under fluoroscopic and endoscopic guidance, a covered colonic SEMS (Niti-S COMVI Enteral Colonic Stent, bare type; TaeWoong Medical, Goyang-si, Korea), 80 mm long and 20 mm in diameter, was successfully deployed to the stenosis (Fig. 3c). The fistula was sealed immediately after placement. Urine culture on day 3 after stenting was negative for bacteria, and a contrast study using water-soluble contrast medium on day 5 confirmed stent patency and showed no fistula (Fig. 3d). The patient was discharged home on day 6 with no complications. The UTI did not recur for 4 months after discharge.

## Discussion

This case highlights two important clinical issues with respect to malignant CVFs. First, a covered colonic SEMS may be useful for nonoperative treatment of a malignant CVF. Second, MRI can be valuable for determining the available treatment strategies for a malignant CVF.

In this case, a covered colonic SEMS was useful for closing a malignant CVF in a patient who required palliative care. The treatment of a malignant CVF is widely dependent on the progression of malignant disease and the general condition of the patient. In patients fit for major surgery, the standard surgical strategy including minimally invasive surgery consists of resection of the involved bowel tract containing the fistula, primary or delayed anastomosis, and closure of the bladder [1, 2, 5]. In patients unfit for major surgery, however, a proximal defunctioning stoma may be the only option that improves the patient’s quality of life [1]. However, this strategy rarely results in fistula closure, and recurrence frequently occurs. In addition, these patients may still be susceptible to recurrent UTI [6]. In the present case, a covered colonic SEMS was placed, because the patient had terminal-stage cancer, his life expectancy was short, and he was unable to manage the stoma on his own. Because the UTI was controlled immediately after stenting and stoma creation was avoided, covered colonic SEMS placement for a malignant CVF may be an alternative option for such surgically unsuitable patients.

A covered SEMS consists of inner and outer nitinol wire meshes with a hydrophobic polytetrafluoroethylene (PTFE) membrane between them and was developed to prevent tumor ingrowth into the stent lumen [7, 8]. In our case, the PTFE membrane acted as a new barrier between the intestinal lumen and the fistula [3, 4], and the stent’s radial force compressed the fistula; these two effects contributed to closure of the fistula. Although there are no clear data on the duration of CVF closure, a PTFE membrane may contribute to long-term fistula closure because of its strong physical and chemical resistance and biocompatibility [4, 7]. A major concern when using covered stents is the risk of stent migration [7, 8]. Stent migration did not occur in this case because in addition to the stenosis as a tumor-related factor, the following features of the stent may have helped prevent migration: the stent was uncovered at both ends (15 mm each), the outer wire meshes were firmly anchored to the tumor, and the oral side had a flared shape [7].

MRI is valuable for determining the treatment strategies for malignant CVFs. In a review of patients with CVFs including benign diseases, computed tomography was considered the gold standard in detecting CVFs, showing diagnostic accuracy up to 90–100%, whereas MRI was suitable for obtaining detailed information of the fistula-forming tissue [1]. In our patient, MRI clearly showed simple morphology with an ~ 15-mm fistula passing through the center of the tumor on T1- and T2-weighted images. Furthermore, T2-weighted images indicated no active inflammation of the surrounding tissue. In addition to these findings, colonoscopy readily provided a frontal view of the tumor, and we determined that fistula closure was technically feasible by routine colonic stenting [9, 10]. Finally, the covered colonic SEMS was successfully placed. However, few reports have focused on the usefulness of covered SEMS for treatment of CVFs [3, 4], and the latest European Society of Gastrointestinal Endoscopy (ESGE) guidelines for colonic stenting do not mention covered SEMS for CVFs [11]. Therefore, it is necessary to accumulate further cases to elucidate the usefulness of covered SEMS for CVFs.

The indication for colonic stenting during antiangiogenic therapy such as BV is controversial. For patients receiving a chemotherapy regimen that includes antiangiogenic therapy, avoidance of colonic stenting is weakly recommended in the ESGE guidelines [11]. In patients already receiving BV, SEMS insertion was shown to be a significant risk factor for complications requiring surgery (hazard ratio, 5.687; 95% confidence interval, 2.372–13.637; *P* < 0.001) [12]. However, Lee et al. [13] reported that the perforation rate was not higher in the BV group (*n* = 104) than in the non-BV group (*n* = 95) (0.9% vs. 3.2%, respectively). Because antiangiogenic therapy plays an important role in preventing increased microvascular density and facilitating the delivery of anticancer agents to the tumor [14], there is a need for more detailed studies of the administration of antiangiogenic therapy to patients with obstructive stage IV colorectal cancer who require colonic stenting.

Fistula closure using covered SEMS has been reported in various gastrointestinal fields. Many such reports are related to the upper gastrointestinal tract, especially the esophagus [15–17], and the ESGE guidelines strongly recommend esophageal covered SEMS placement for sealing malignant tracheoesophageal or bronchoesophageal fistulas [18]. In addition, the usefulness of SEMS placement for colovaginal fistulas has been reported. Lamazza et al. [19] found that covered SEMS placement was effective in rectovaginal fistulas, with fistula closure possible in 12 of 14 (85.7%) patients. By contrast, no reports have demonstrated the utility of covered SEMS placement for fistula formation caused by diverticulitis or Crohn’s disease. Therefore, although covered SEMS placement may be a useful technique for fistula closure, careful patient selection, including detailed imaging studies, is vital for its application because stent-related complications such as perforation are often life-threatening [11, 20–22].

## Conclusions

A covered colonic SEMS was useful for a malignant CVF in a patient unfit for surgery, and MRI was valuable to determine the status of the fistula. Avoidance of stoma creation in the terminal stage of cancer is important for not only maintaining quality of life but also reducing the psychological burden. Because of stent-related complications (e.g., perforation, migration, and bleeding) and the lack of evidence regarding stenting for CVF, surgical treatment is clearly the first choice for patients who can tolerate major surgery. However, a covered colonic SEMS may be an alternative to surgical treatment of malignant CVF in patients who require palliative care.

## Data Availability

All data analyzed in this study are included in this manuscript.

## Abbreviations

BV: Bevacizumab
CVF: Colovesical fistula
MRI: Magnetic resonance imaging
PTFE: Polytetrafluoroethylene
SEMS: Self-expanding metallic stent
UTI: Urinary tract infection
